# Editorial: The Influence of Loud Music on Physical and Mental Health

**DOI:** 10.3389/fpsyg.2019.02149

**Published:** 2019-09-19

**Authors:** Mark Reybrouck, Piotr Podlipniak, David Welch

**Affiliations:** ^1^Musicology Research Group, Faculty of Arts, KU Leuven-University of Leuven, Leuven, Belgium; ^2^IPEM, Department of Art History, Musicology and Theatre Studies, Ghent, Belgium; ^3^Institute of Musicology, Adam Mickiewicz University in Poznań, Poznań, Poland; ^4^Audiology Section, School of Population Health, University of Auckland, Auckland, New Zealand

**Keywords:** hearing loss, loud music, noise annoyance, biological stressors, biomarkers, CAALM model

Music and noise can be considered as a collection of vibrational events which may impinge upon the body and the mind. As such they can induce beneficial or harmful bodily and psychological reactions. Much contemporary music production and consumption, however, produces sensory saturation and/or overload with sounds being manipulated in terms of spectrum and dynamic range. Such manipulation is not harmful by definition, but the manipulations may increase the potential for harm. Much research has been devoted to the risk of auditory overstimulation, but the topic is still relevant as listening to music is a significant form of leisure noise exposure. Listening habits and attitudes toward loud music may even promote behaviors that could cause harm to listeners, both in a direct and indirect way. Listening to loud music, in fact, may be damaging to the cochlea (and possibly also the auditory nuclei and the auditory pathway), and may cause both temporary or permanent hearing loss. On the other hand, loud music may have benefits for us, particularly in terms of social integration and an enhanced sense of wellbeing and fun. Several questions can therefore be raised. What are the physical effects of sound as transferable and vibrational energy? Are there constraints and biases that shape and modify our reactions to the sounds? What are the perceptual and associated behavioral aspects? What are the psychological mechanisms for liking loud music? What are the social factors that contribute to increased preferences for loud music? And how can we assess the beneficial and harmful effects of sounds that are outside of the optimal range of stimulation?

With a view to answer these questions, this special Research Topic on “The Influence of Loud Music on Physical and Mental Health” has been launched. Contributions have been solicited from leading authors in the fields of acoustics, psychoacoustics, audiology, sensory biology, neurosciences, psychology, and sociology with the aim to provide a view of the state of the art about the effects of loud music on human beings. In order to provide a broader overview, care has been taken not to focus exclusively on possible harmful effects, but also to investigate the underlying mechanisms that bring people to risky listening behaviors, even when they know that loud music may be harmful for their auditory system and their wellbeing in general.

The answer to the call for papers was not really abundant, which is strange, given the importance of the dangers of maladaptive listening which is quite common in the music consumption by many young listeners nowadays.

Five papers have been selected finally for publication in this Research Topic. They can be divided into theoretical and empirical papers, which focus mainly on five major domains: (i) the major constituting and characterizing features of hazardous sounds, (ii) the effects of loud music on biological organisms, (iii) the underlying mechanisms of hearing loss or damage to the auditory system, (iv) sociocultural, psychological, and behavioral aspects of listening to loud and noisy music, and (v) the use of loud music to boost performance in sports and exercise.

The contribution by Reybrouck et al. provides a general overview of the effects of loud music and noise on our body. Starting from a conception of music in terms of vibrational and transferable energy, it investigates two levels of description: the physical-acoustical description of the sounds and the subjective-psychological reactions by the listener. It investigates how sound may activate the sense of touch and the vestibular system of the inner ear besides the sense of hearing. It then elaborates on the direct and indirect effects of loud music and noise and delves into the widespread phenomenon of liking loud music from a behavioral and psychological view. Alvarado et al. take a more empirical stance toward the main topic by studying the impact of repeated short-duration overexposure on Wistar rats. By means of auditory brainstem response recordings they were able to demonstrate a significant increase in auditory thresholds, due to decreased cochlear inputs, impaired afferents in auditory nuclei and impaired neurotransmission along the auditory pathway, and state that age-related hearing loss is accelerated by such sound stimulation. In an online hearing survey Beach and Gilliver did an empirical study of the preference of loudness levels of regular patrons of music venues. By examining their attitudes and preferences toward sound levels and protective listening behaviors they found out that the majority of patrons was dissatisfied with current sound levels. Venues should be motivated accordingly to meet their patrons' needs and preferences by providing sound levels that are not louder than desirable for the majority of patrons. In her study in the domain of sports and exercise Van Dyck investigated whether musical intensity is an effective strategy for boosting performance. It has been shown that musical intensity may increase running, grip strength, and choice reaction time, while decreasing simultaneously time to exhaustion and level of perceived exhaustion. There is, however, still scarce evidence for these claims, with much variation in the experimental designs and tested groups and a lot of contradicting evidence. Though there are some benefits, sports and exercise listeners fail to consider high-intensity music to be too loud if they find it enjoyable or motivating. The contribution by Manchaiah et al., finally, investigates young adults' knowledge and attitude regarding loud music in distinct countries. They criticize some attitude studies as there is little relation between the expressed attitude and behavior and explore the social representations of loud music by relying on the social representations theory. The latter seems to be more fundamental than attitude as it has a better relation to behavior. In their study they present an overview and discussion.

## Author Contributions

All authors listed have made a substantial, direct and intellectual contribution to the work, and approved it for publication.

### Conflict of Interest

The authors declare that the research was conducted in the absence of any commercial or financial relationships that could be construed as a potential conflict of interest.

